# Effect of coffee or coffee components on gut microbiome and short-chain fatty acids in a mouse model of metabolic syndrome

**DOI:** 10.1038/s41598-018-34571-9

**Published:** 2018-11-01

**Authors:** Kazuchika Nishitsuji, Syunsuke Watanabe, Jinzhong Xiao, Ryosuke Nagatomo, Hirohisa Ogawa, Takaaki Tsunematsu, Hitomi Umemoto, Yuki Morimoto, Hiroyasu Akatsu, Koichi Inoue, Koichi Tsuneyama

**Affiliations:** 1 Department of Pathology and Laboratory Medicine, Graduate School of Biomedical Sciences, Tokushima University, 3-18-15 Kuramoto-cho, Tokushima, 770-8503 Japan; 2 Department of Biochemistry, Wakayama Medical University, 811-1 Kimiidera, Wakayama, 641-8509 Japan; 3 Next Generation Science Institute, Morinaga Milk Industry Co., Ltd., 5-1-83 Higashihara, Zama, Kanagawa 252-8583 Japan; 4 Laboratory of Clinical and Analytical Chemistry, College of Pharmaceutical Sciences, Ritsumeikan University, 1-1-1 Nojihigashi, Kusatsu, Shiga 525-8577 Japan; 5 Education Support Room for Anatomy, Tokushima University, 3-18-15 Kuramoto-cho, Tokushima, 770-8503 Japan; 6 Department of Medicine for Aging in Place and Community-Based Medical Education, Nagoya City University, Graduate School of Medical Sciences, 1 Kawasumi, Mizuho-cho, Mizuho-ku, Nagoya, Aichi 467-8601 Japan

## Abstract

We previously showed that male Tsumura Suzuki obese diabetes (TSOD) mice, a spontaneous mouse model of metabolic syndrome, manifested gut dysbiosis and subsequent disruption of the type and quantity of plasma short-chain fatty acids (SCFAs), and daily coffee intake prevented nonalcoholic steatohepatitis in this mouse model. Here, we present a preliminary study on whether coffee and its major components, caffeine and chlorogenic acid, would affect the gut dysbiosis and the disrupted plasma SCFA profile of TSOD mice, which could lead to improvement in the liver pathology of these mice. Three mice per group were used. Daily intake of coffee or its components for 16 wk prevented liver lobular inflammation without improving obesity in TSOD mice. Coffee and its components did not repair the altered levels of Gram-positive and Gram-negative bacteria and an increased abundance of Firmicutes in TSOD mice but rather caused additional changes in bacteria in six genera. However, caffeine and chlorogenic acid partially improved the disrupted plasma SCFA profile in TSOD mice, although coffee had no effects. Whether these alterations in the gut microbiome and the plasma SCFA profile might affect the liver pathology of TSOD mice may deserve further investigation.

## Introduction

Metabolic syndrome is a disorder that encompasses a group of symptoms that are related to obesity and metabolic modifications, and raises the risk of developing cardiovascular disease and type 2 diabetes mellitus^1,2^. This syndrome has the clinical manifestations of hyperglycemia, insulin resistance, hyperlipidemia, nonalcoholic fatty liver disease (NAFLD), and progressive phenotype of NAFLD, nonalcoholic steatohepatitis (NASH)^3–5^. The crucial characteristic of metabolic syndrome is persisting low-grade inflammation^6^. We previously showed that Tsumura Suzuki obese diabetes (TSOD) mice, that were found to spontaneously develop type 2 diabetes mellitus in the original reports^7,8^, also spontaneously developed NASH^9^. Currently, TSOD mice are an accepted mouse model of metabolic syndrome^10^.

Mammals possess diverse and extremely active gut microbiota that includes over 10 trillion microbial cells and 1000 microbial strains^11^. Gut microbiota affects the physiology of the hosts ranging from energy metabolism to immune responses^12,13^, and growing evidence supports that alterations in gut microbiota composition, i.e., changes that are referred to as gut dysbiosis, involve in the development of metabolic syndrome including NAFLD, NASH, and diabetes mellitus especially from the aspect of inflammation that is associated with obesity^14–26^. The most plentiful product of undigested dietary fibers by bacterial fermentation are 1–6 carbons-containing short-chain fatty acids (SCFAs) which link gut microbiota and the host’s physiology^12,27–29^. In addition to functioning as an energy substrate, SCFAs affect several physiological processes of the hosts’ tissues and organs by modulating anti-inflammatory responses and neuroendocrine system^29,30^. Thus, the gut microbiota plays a critical role in the development of obesity and metabolic syndrome via regulating the types and quantity of SCFAs. Indeed, we previously reported that metabolic syndrome-affected TSOD mice demonstrated gut dysbiosis and subsequent disruption of the plasma SCFA profile^31^.

Coffee is one of the most popular beverages worldwide. It contains more than 1500 chemical components including phenolic polymers, polysaccharides, minerals, caffeine, and chlorogenic acid^32^. In addition to caffeine, which is a major water-soluble component of coffee (1%), several other components such as chlorogenic acid (4%) are reportedly biologically active^33^. Consumption of coffee or chlorogenic acid has been associated with alterations in the risk of metabolic syndrome and NAFLD^34,35^. Also, we previously reported that daily intake of coffee, not caffeine-free coffee, prevented the onset of hepatic pathology without affecting obesity or hyperlipidemia in TSOD mice^36^. Here, we focused on caffeine and chlorogenic acid as major bioactive coffee components, and we hypothesized that coffee or its major components may improve the hepatic pathology in TSOD mice by repairing the gut dysbiosis and disrupted plasma SCFA profile.

## Results

### Daily intake of coffee or major coffee components prevented hepatic lobular inflammation in TSOD mice

**Figure 1:**
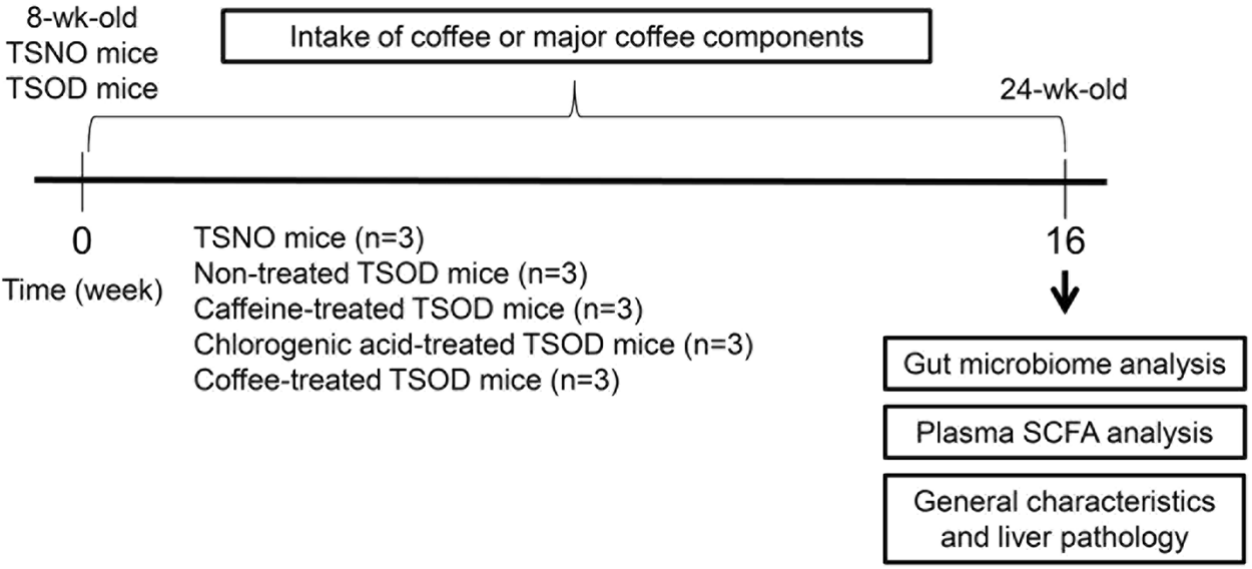
Experimental flow chart. Animals were divided into five groups (n = 3 in each group): TSNO mice; non-treated TSOD mice; caffeine-treated TSOD mice; chlorogenic acid-treated TSOD mice; and coffee-treated group. After the experiment, the liver was subjected to histopathological and immunohistochemical analysis.

**Figure 2:**
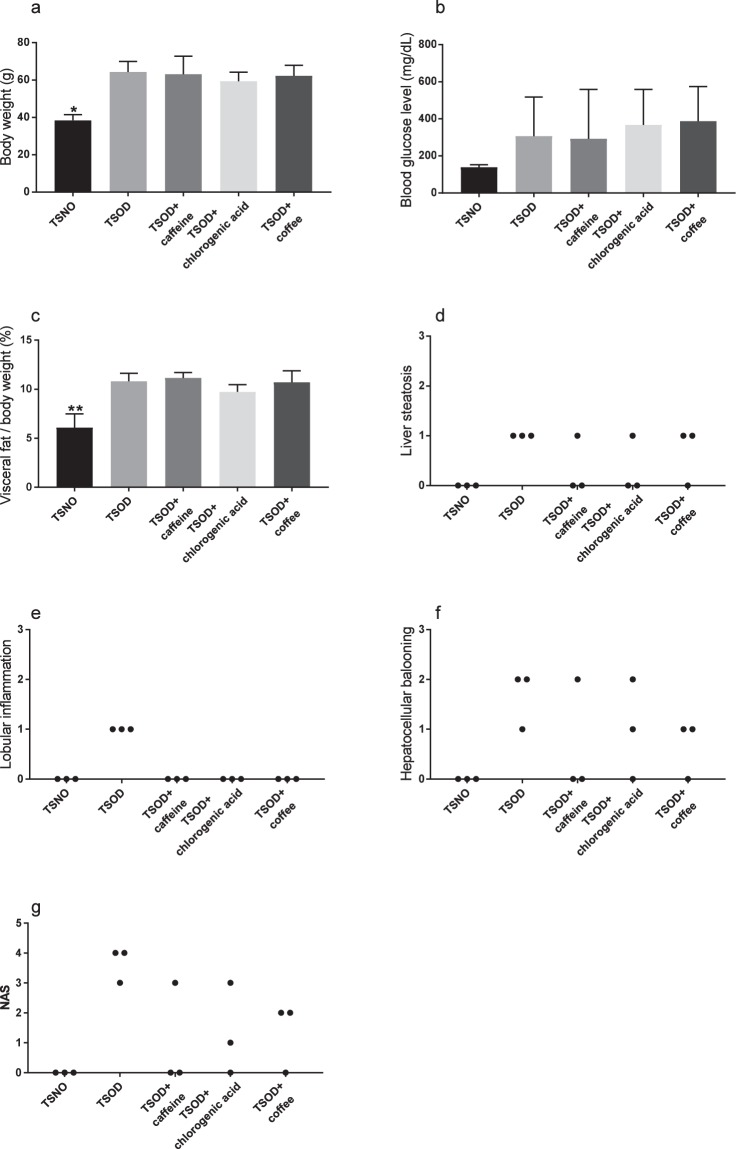
General liver characteristics and pathological scores in 24-wk-old TSOD mice that were treated with coffee or its components and in age-matched TSNO mice. (**a**) Body weight (**b**) blood glucose level (**c**) ratio of visceral fat to body weight, and scores for (**d**) liver steatosis (**e**) lobular inflammation (**f**) hepatocellular ballooning and (**g**) NAFLD activity score (NAS). Data are means ± SD. *P < 0.05; **P < 0.01 versus all groups.

The experimental design of this study is shown in Fig. 1. We first analyzed the general characteristics and histopathology of the livers of 24-wk-old TSOD male mice that were given caffeine, chlorogenic acid, or coffee and age-matched Tsumura Suzuki non-obesity mice (TSNO mice, controls). As Fig. 2a,c shows, 24-wk-old TSOD mice demonstrated obesity-related alterations, such as increased body weight (1.5-fold increase, *P* < 0.01) and visceral fat (1.5-fold increase, *P* < 0.01), compared with TSNO mice. We did not detect significant differences in blood glucose levels in TSNO and TSOD mice (Fig. 2b). These obesity-related alterations were not improved by the intake of coffee or its components (Fig. 2a,c). The histopathological characteristics of the liver were scored according to our previous report^9^ (Fig. 2d–f). The average of NAS scores of TSOD mice were 3.7, which was comparable to those in our previous report (Fig. 2g)^9^. Liver lobular inflammation, i.e., infiltration of inflammatory cells into liver parenchyma, was reduced in the coffee-, caffeine-, and chlorogenic acid-treated groups (Fig. 2d).

### The gut microbiome in TSOD mice that were treated with coffee or its components

**Figure 3:**
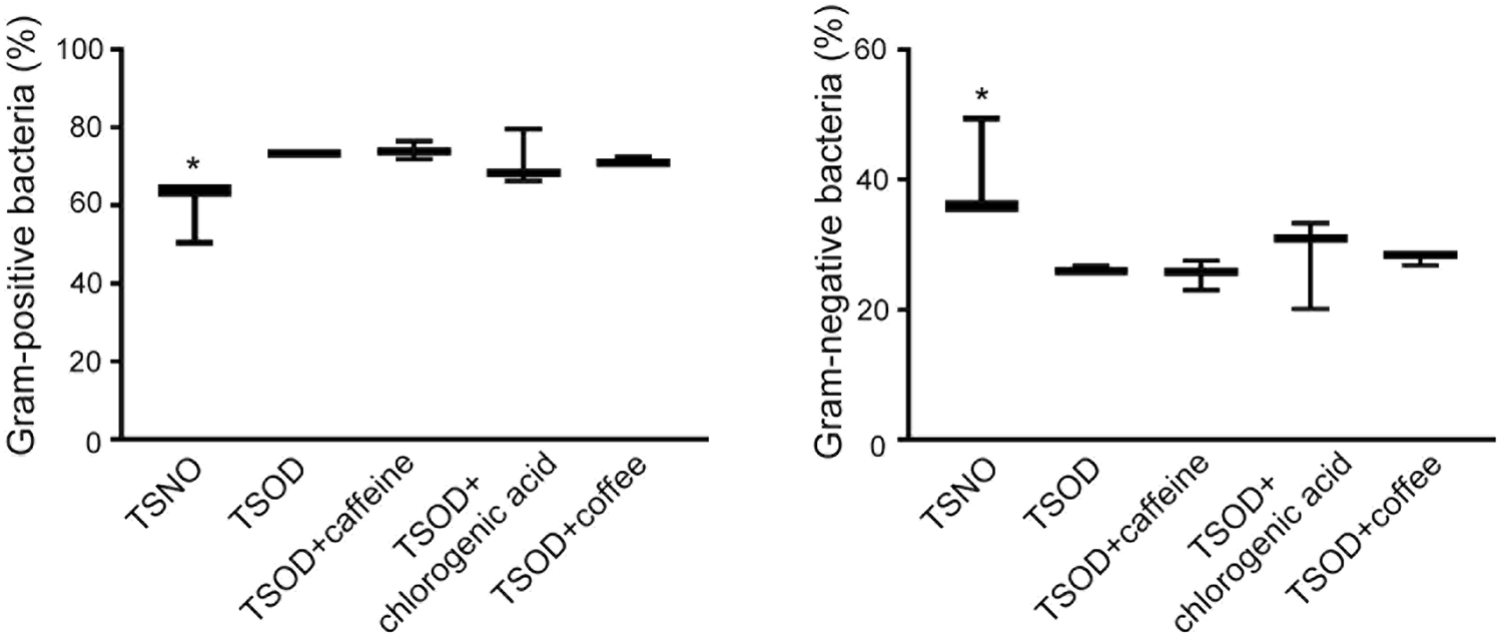
Analysis of the percentages of Gram-positive bacteria and Gram-negative bacteria in 24-wk-old TSOD mice that were treated with coffee or its components and in age-matched TSNO mice. Comparison of the percentages of Gram-positive bacteria and Gram-negative bacteria. Boxes show the interquartile ranges of the first and third quartiles; medians are shown by the lines in the boxes. If no error bars are shown, the experimental error was smaller than the symbol. *FDR adjusted *Q* < 0.05 versus non-treated TSOD, TSOD + caffeine and TSOD + coffee mice.

**Figure 4:**
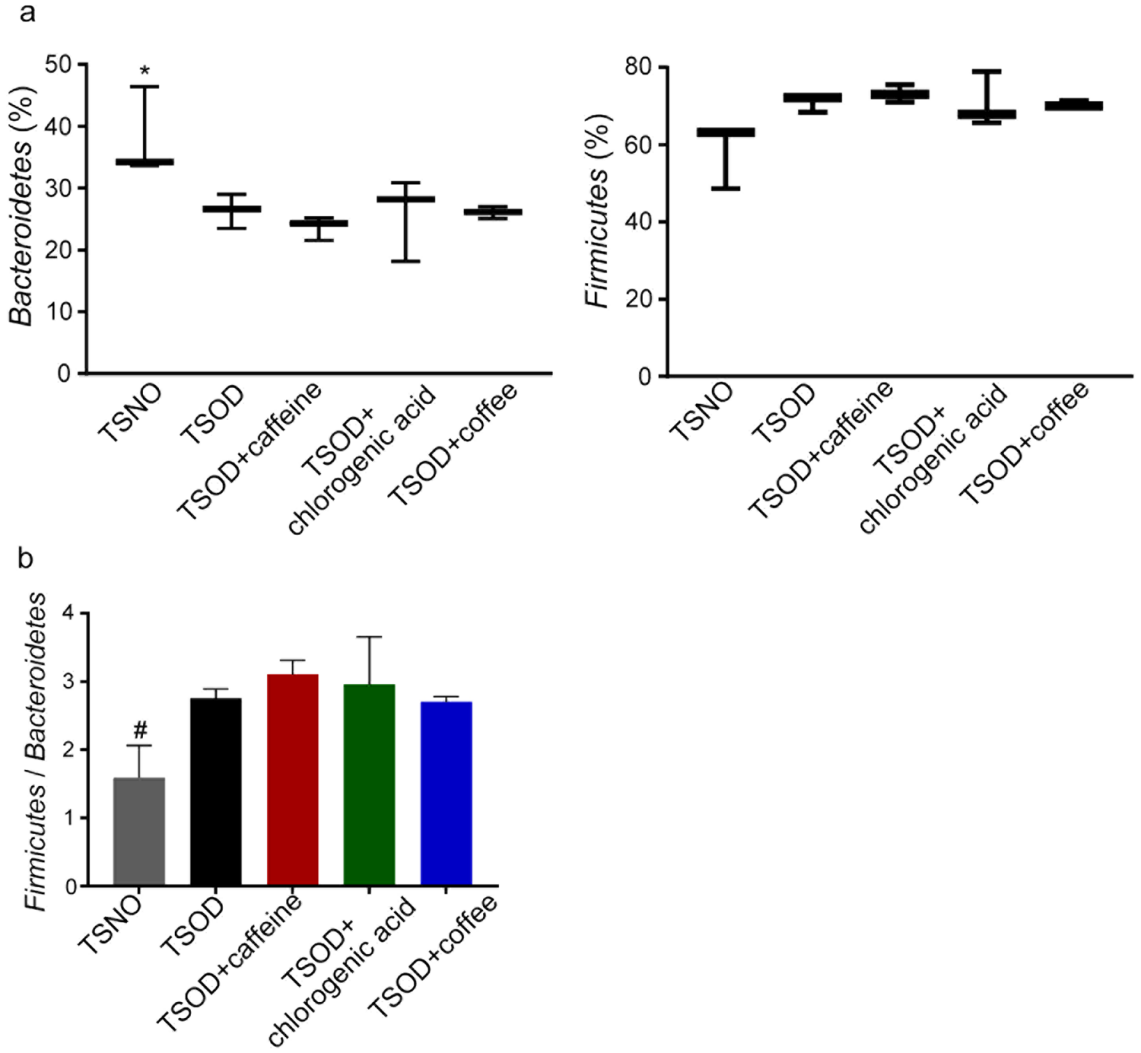
Analysis of fecal bacteria at the phylum level in caffeine-, chlorogenic acid-, and coffee-treated groups and in age-matched TSNO mice. (**a**,**b**) Comparison of the percentages (**a**) and ratio (**b**) of the Firmicutes bacteria and the Bacteroidetes bacteria in caffeine-, chlorogenic acid-, and coffee-treated groups. (**a**) Boxes show the interquartile ranges of the first and third quartiles; medians are shown by the lines in the boxes. If no error bars are shown, the experimental error was smaller than the symbol. *FDR adjusted *Q* < 0.05 versus non-treated TSOD mice. (**b**) Data are means ± SEM (n = 3). ^#^*P* < 0.05 versus non-treated TSOD, TSOD + caffeine and TSOD + chlorogenic acid mice.

The use of fecal microbiota as a substitute for gut microbiota is generally accepted, so we analyzed the species in the microbiota via 16S ribosomal RNA (rRNA) gene sequencing of DNA obtained from the fecal samples. As Fig. 3 illustrates, 24-wk-old metabolic syndrome mice (TSOD mice) and age-matched TSNO mice (controls) had different levels of Gram-positive (1.2-fold increase, FDR adjusted *Q* < 0.05) and Gram-negative bacteria (0.8-fold decrease, FDR adjusted *Q* < 0.05), which is consistent with results found in our previous report^31^. However, we observed no significant changes in the contents of Gram-positive and Gram-negative bacteria among the untreated TSOD mice and the coffee- or coffee component-treated TSOD mice (Fig. 3). As in our previous report, we found a 1.1-fold increased percentage of Firmicutes with a favorable trend (FDR adjusted *Q* = 0.0594) and a significant decrease in Bacteroidetes (0.8-fold decrease, FDR adjusted *Q* < 0.05) in TSOD mice compared with TSNO mice (Fig. 4a)^31^. The ratio of Firmicutes to Bacteroidetes was 1.6-fold higher in TSOD mice compared to TSNO mice (*P* < 0.05, Fig. 4b). Again, we observed no changes in the percentages of these bacteria (Fig. 4a,b). These results suggest that daily intake of coffee or its components did not repair the gut dysbiosis in TSOD mice.

Instead, in the fecal samples from the mice, we found that the percentages of six genera changed significantly versus non-treated TSOD mice in the groups treated with caffeine, chlorogenic acid, or coffee (Table 1). These bacteria included *Blautia*, *Coprococcus*, and *Prevotella*, which have been implicated in inflammation or obesity^37–43^.

**Table 1:** Bacteria whose percentages changed significantly in 24-wk-old TSOD mice that were treated with caffeine, chlorogenic acid, or coffee.

Phylum	Class	Order	Family	Genus	TSNO	TSOD	TSOD + caffeine	TSOD + chlorogenic acid	TSOD + coffee
Actinobacteria	Actinobacteria	Actinomycetales	Corynebacteriaceae	*Corynebacterium*	0	0	0.000795	0	0
					0–0	0–0	0.000745–0.002314*	0–0.000227	0–0
Firmicutes	Bacilli	Bacillales	Staphylococcaceae	*Jeotgalicoccus*	0	0.0000394	0.001192	0	0
					0–0	0–0.00008425	0.000883–0.00217*	0–0.000426	0–0.000423
Firmicutes	Bacilli	Lactobacillales	Aerococcaceae	*Facklamia*	0	0	0.001302	0.000194	0
					0–0	0–0	0.000298–0.001325*	0.000114–0.000851	0–0
Firmicutes	Clostridia	Clostridiales	Lachnospiraceae	*Blautia*	0.000728	0.000281	0.000447	0.001589	0.000317
					0.0001965–0.001801	0.0001435–0.000363	0.000289–0.001237	0.000872–0.002894*	0–0.00053
Firmicutes	Clostridia	Clostridiales	Lachnospiraceae	*Coprococcus*	0.005388	0.004505	0.006094	0.01347	0.003339
					0.002686–0.006538	0.001799–0.01095	0.004618–0.008535	0.01283–0.01439*	0.002009–0.004507
Bacteroidetes	Bacteroidia	Bacteroidales	Prevotellaceae	*Prevotella*	0.004087	0.004174	0.002031	0.001447	0.0007402
					0.002854–0.006105	0.002591–0.00574	0.001736–0.002681	0.001022–0.002906	0.0006629–0.001669**

Values are medians and interquartile ranges of the percentages. FDR adjusted *Q*-value of 0.05 was considered statistically significant. *Q* values versus untreated TSOD mice were calculated by the Benjamini and Hochberg method and the results were considered statistically significant when *Q* < 0.05. *Q < 0.05, **Q < 0.01 versus non-treated TSOD mice.

### The type and quantity of SCFAs in plasma

**Figure 5:**
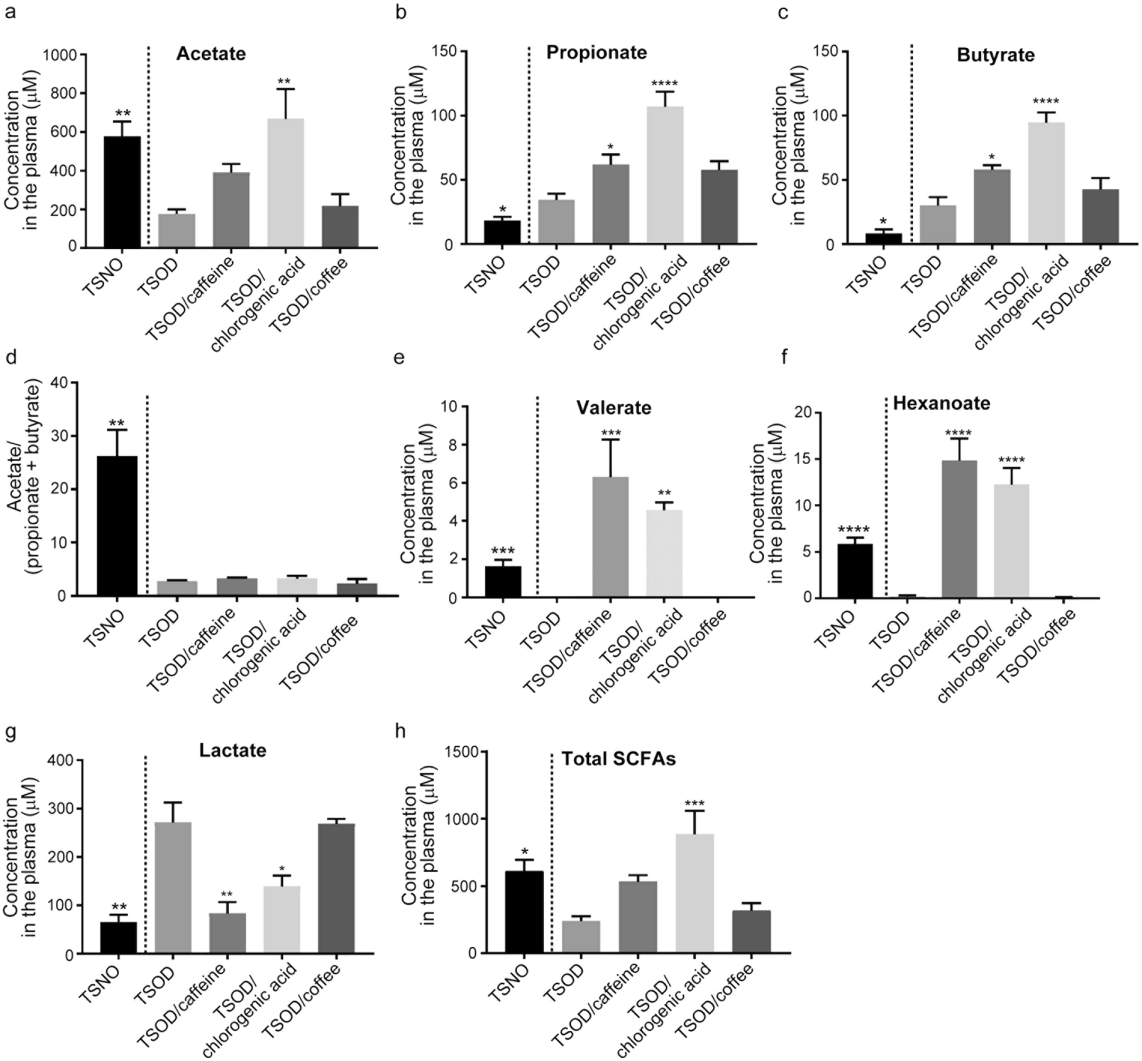
Analysis of plasma SCFAs in caffeine-, chlorogenic acid-, and coffee-treated groups and in age-matched TSNO mice. (**a**–**d**) Concentrations of the major SCFAs (**a**, acetate; **b**, propionate; and **c**, butyrate) and the ratio of acetate to propionate plus butyrate (**d**). (**e**–**h**) The concentrations of the minor SCFAs (**e**, valerate; and **f**, hexanoate) and the concentration of total plasma SCFAs (**h**). The Concentration of the precursor of SCFAs, lactate, was also determined (**g**). Data are means ± SEM (n = 3). *P < 0.05; **P < 0.01; ***P < 0.001; ****P < 0.0001 versus non-treated TSOD mice.

In our previous study, we reported dysbiosis and disturbances in the type of plasma SCFAs in metabolic syndrome-affected TSOD mice^29^. Thus, we next investigated the effects of daily consumption of coffee or its components on plasma SCFA profiles in TSOD mice. Figure 5a indicates that chlorogenic acid recovered the reduced acetate level in TSOD mice. However, caffeine and chlorogenic acid increased the plasma concentrations of propionate and butyrate even more (propionate, 1.5- to 4-fold increase, *P* < 0.05 (caffeine-treated group) and *P* < 0.0001 (chlorogenic acid-treated group) versus non-treated group; butyrate, 1.5- to 3-fold increase, *P* < 0.05 (caffeine-treated group) and *P* < 0.0001 (chlorogenic acid-treated group) versus non-treated group; Fig. 5b,c). The ratio of acetate to butyrate plus propionate in TSOD mice therefore did not change in the caffeine- and chlorogenic acid-treated groups (Fig. 5d). The levels of the minor SCFAs valerate and hexanoate, which were almost not measurable in untreated TSOD mice, reached detectable levels in the caffeine-treated and chlorogenic acid-treated groups (Fig. 5e,f). The quantity of lactate, the precursor of SCFAs^29^, was significantly reduced in the caffeine-treated and chlorogenic acid-treated groups (0.3- to 0.5-fold decreases, *P* < 0.01 (caffeine-treated group) and *P* < 0.001 (chlorogenic acid-treated group) versus non-treated group, Fig. 5g). To our surprise, however, we found no significant changes in the type and quantity of plasma SCFAs in the coffee-treated group (Fig. 5a–h). In addition, the plasma concentration of lactate in the coffee-treated group was similar to that in the non-treated TSOD group (Fig. 5g).

## Discussion

Metabolic syndrome significantly alters gut microbiota in humans and in animal models. The most prominent changes are a reduced abundance of Bacteroidetes that is accompanied by an increased occurrence of Firmicutes in obese mice^19^, humans^17^, and patients with NASH^21^. We also previously reported an increased occurrence of Firmicutes species together with a corresponding decrease in Bacteroidetes species in TSOD mice^31^. In addition, we reported that the percentages of several bacteria at the family and genus levels were altered in TSOD mice, a spontaneous model of metabolic syndrome, which led us to conclude that TSOD mice had their own microbial signature, called the TSOD microbiome. As an important finding, this TSOD microbiome occurred independently of diet, which is consistent with findings in the report of Ley *et al*.^19^ and suggests that the TSOD microbiome was related to metabolic syndrome.

Because of our previous report showing that coffee and its components prevented the development of NASH pathological characteristics in TSOD mice^36^, for this study we investigated the hypothesis that coffee or its components can affect the gut microbiome and SCFA profile in TSOD mice and thereby improve hepatic inflammation. Here, caffeine and chlorogenic acid treatments partly restored the disrupted plasma SCFA profile in TSOD mice. Because we fed all mice standard chow throughout the study, the determinant of the SCFA profile was presumably the composition of the gut microbiota in these groups. Thus, our results suggest that although the effect of consumption of caffeine or chlorogenic acid on the gut microbiome is not accompanied by marked changes, these coffee components might repair the disrupted SCFA profile by modulating the gut microbial community. Additional experiments with a number enough of mice to allow possible correlations would be needed to evaluate this possibility.

Daily intake of coffee or its components did not improve the gut dysbiosis in TSOD mice. Rather, we found that the percentages of six microbial genera changed in these mice. These genera were completely different from genera that were altered in TSOD mice in our previous report^31^. Among them, we found that the percentages of *Blautia* and *Coprococcus* in the Firmicutes phylum were higher in the chlorogenic acid-treated group than in the untreated group. The percentage of bacteria in these genera reportedly increased in obese individuals in a Japanese population^44^. Another study also reported an increased occurrence of *Blautia* in rats fed a high-fat diet^37^. These data suggest a potential role of these genera in the development of obesity. However, the occurrence of *Coprococcus* correlated with fasting levels of gastrointestinal polypeptide in obese women^38^. Because gastrointestinal polypeptide is known to stimulate insulin secretion^39^, *Coprococcus* may protect against metabolic syndrome. The percentage of *Blautia* bacteria reportedly increased in humans after consumption of whole grain, which may contribute to the anti-inflammatory effect of whole grain^40^. As an intriguing result, the daily intake of chlorogenic acid in the present study improved the hepatic lobular inflammation of TSOD mice without altering the body weight. Because *Blautia* was reportedly involved in acetate production^45^, the increased abundance of *Blautia* may contribute to the increased plasma concentration of acetate. However, given that the plasma SCFA profile depends on the whole microbial community^30^, identifying a single bacterial species responsible for the alteration of the quantity or type of plasma SCFAs in caffeine-treated and chlorogenic acid-treated groups will be difficult. The involvement of *Prevotella* species, which are Gram-negative rods, is relatively well-documented. A decrease in the percentage of *Prevotella* species was associated with improvement in endotoxemia and systemic inflammation in diabetic mice^42^. The abundance of *Prevotella* also reportedly increased in obese humans^43^. In agreement with these reports, the percentage of *Prevotella* decreased in our coffee-treated TSOD mice. As *Prevotella* has been implicated in degradation of dietary fibers^46^, the abundance of this bacteria might depend on the brand of coffee. Involvement of these genera in Table 1 in the pathology of metabolic syndrome remains to be elucidated.

The mechanisms by which daily consumption of coffee improved liver lobular inflammation remain to be clarified. Although coffee contains caffeine and chlorogenic acid, daily consumption of coffee did not fix the disrupted TSOD mouse-specific SCFA profile. The altered gut microbiota at the genus level in the coffee-treated group could contribute to the improvement in lobular inflammation, but we cannot exclude the possibility that pathways other than those mediated by gut microbiota also contributed to the protective effects of coffee. Several coffee components reportedly act as antioxidants by scavenging free radicals,^32,34^ which may promote the development of NAFLD^47,48^. Furthermore, coffee reduced hepatic concentrations of proinflammatory cytokines such as tumor necrosis factor α and interferon γ and increased those of anti-inflammatory cytokines such as interleukins 4 and 10 in an animal model of steatohepatitis, which led to reduced liver damage^49^. Caffeine also has hepatoprotective effects^50,51^. These lines of evidence strongly suggest that coffee reduced liver lobular inflammation via multiple pathways. Coffee includes dietary fibers that can be fermented by gut microbiota to produce acetate, propionate, and butyrate^52^. However, in the present study, we observed no alterations in the type and quantity of plasma SCFAs in the coffee-treated group. Because composition of coffee differs by brand, this discrepancy might be due to the coffee brand used.

In summary, we showed that coffee and its components improved liver lobular inflammation in a spontaneous mouse model of metabolic syndrome, affected also the gut microbial community and, in the case of the coffee components additionally influenced plasma SCFA profile. Some of the bacterial genera identified could be further studied as potentially protective against the pathology of metabolic syndrome particularly that is associated with inflammation. Additional studies with more animals in each group and a longer consumption period are necessary to investigate the effects of coffee and its components on various pathological features of metabolic syndrome. Studies of patients are also a challenge for the future.

## Methods

### Animals

Male twelve TSOD mice and three TSNO mice were purchased from the Institute for Animal Reproduction (Ibaraki, Japan). We reared two or three mice in plastic cages that were kept in a non-barrier-maintained animal room under the following conditions: 23 ± 2 °C, 50 ± 10% relative humidity, and a 12/12-h light/dark cycle. All mice were fed the basal diet MF (Oriental Yeast Co., Ltd., Tokyo, Japan) and had access to chlorinated water *ad libitum*. Eight-week-old TSOD mice were given coffee (Nescafe Gold Blend Gold Label; Nestle Japan, 0.5%) or its components, caffeine (INDOFINE Chemical Company, Inc., Hillsborough, NJ, 0.012%) or chlorogenic acid (INDOFINE Chemical Company, Inc., 0.0195%), by mouth, for 16 wk (referred to as the coffee-treated group, caffeine-treated group, and chlorogenic acid-treated group, respectively). Each group contained three mice. The content of caffeine or chlorogenic acid corresponded to that in 0.5% coffee. Coffee and its components were administered by dissolving the test substance in self-pumped water containing sodium hypochlorite. The animals had free access to these liquids. No differences were noted in the volume of liquids that the animals in the different groups drank throughout the study. The intake amounts of coffee and its components in the present study fell within the range of amounts recommended by the European Food Safety Authority. This study was performed according to the “Consensus Author Guidelines on Animal Ethics and Welfare” specified by the Institute for Animal Reproduction (Ibaraki, Japan), which were developed and published in 2010 by the International Association of Veterinary Editors. All experimental protocols were also approved by the animal research committee of the Institute for Animal Reproduction.

### Analysis of the gut microbiome

We used the isopropanol precipitation technique to extract DNA from fecal samples. In this method, we suspended mouse feces (30–40 mg) in phosphate-buffered saline (19× volume) and homogenized the sample with the FastPrep-24 homogenizer (MP Biomedicals, Santa Ana, CA). A sample that contained 250 μL of TE buffer (200 mM Tris-HCl, 80 mM ethylenediaminetetraacetic acid, pH 9.0), 500 µL of TE-saturated phenol (Nippon Gene Co., Ltd., Tokyo, Japan), 50 µL of 10% sodium dodecyl sulfate, and 0.3 g of glass beads (0.1 mm diameter; As-One Co., Ltd., Osaka, Japan, #BZ-01) was added to 200 μL of the homogenized samples of feces. These samples were homogenized again for 30 s with the FastPrep-24 homogenizer, and then they were centrifuged at 15,000 rpm at 4 °C for 5 min. We added a 400-μL sample of a mixture of phenol, chloroform, and isoamyl alcohol (25:24:1) (Nippon Gene Co., Ltd.) to the supernatant, vortexed it for 10 s, and centrifuged it at 15,000 rpm at 4 °C for 5 min. We added 250 μL of isopropanol obtained from Wako Pure Chemical Industries Ltd. (Osaka, Japan) to 250 μL of the supernatant, mixed it by flipping, and kept the mixture at room temperature for 10 min, after which we centrifuged the sample at 15,000 rpm at room temperature for 10 min. We removed the supernatant and washed the resultant pellet with 400 μL of ice-cold ethanol. We air-dried the extracted DNA and then dissolved it in 2000 μL of TE buffer (pH 8.0). We amplified the V3-V4 region of the bacterial 16 S rRNA gene by means of PCR with the TaKaRa Ex Taq HS Kit (TaKaRa Bio, Shiga, Japan) and the Tru357F primer set (5ʹ-CGCTCTTCCGATCTCTGTACGGRAGGCAGCAG-3ʹ) and Tru806R primer set (5ʹ-CGCTCTTCCGATCTGACGGACTACHVGGGTWTCTAAT-3ʹ). We concentrated the DNA by amplifying it, in triplicate, via PCR: 94 °C preheating for 3 min, and then 30 cycles of denaturation at 94 °C for 30 s, annealing at 50 °C for 30 s, extension at 72 °C for 30 s, and a terminal extension at 72 °C for 5 min. We then amplified the 16 S rRNA gene amplicons with a 2nd primer set that was adapted for the Illumina MiSeq (Illumina, San Diego, CA) as previously described^53^. DNA was amplified according to the above-mentioned thermal cycling pattern, except that the cycle number was 15, and the 2nd amplified PCR products were purified by using a QIAquick PCR Purification Kit (Qiagen, Hilden, Germany), after which purified PCR products were quantified with a Quant-iT PicoGreen dsDNA Assay kit (Thermo Fisher Scientific, Carlsbad, CA). After removing primer-dimer by using the QIAquick PCR Purification Kit, sequences of the libraries were determined by means of an Illumina MiSeq instrument with a MiSeq v3 Reagent kit (Illumina, San Diego, CA). Data were analyzed as previously described^54^ with several modifications. The Illumina paired-end reads that passed the quality filters were combined by using the fastq-join script in ea-utils (ver. 1.1.2–537)^55^. Potential chimeric sequences were excluded by using reference-based chimera checking in USEARCH (ver. 5.2.32)^56^ and the gold database (http://drive5.com/otupipe/gold.tz). Non-chimeric sequences were analyzed with the QIIME software package (version 1.7.0^57,58^) by using closed-reference operational taxonomic unit (OTU) picking against 16S rRNA genes of 15 species of predominant human gut-derived microbiota and *B. longum* BB536.

### Histopathological and immunohistochemical analyses

We recorded the body weights at the start and at the end of the experimental period. Immediately after the animals were killed, we rapidly excised the liver and visceral fat and rinsed them in ice-cold saline. We fixed the excised organs with 10% neutral-buffered formalin and embedded them in paraffin for histological analysis. We stored some liver samples at −80 °C. We cut formalin-fixed, paraffin-embedded liver tissue into 4-μm-thick serial sections and stained them with hematoxylin and eosin. We also stained a frozen 5-μm-thick section with Sudan IV and hematoxylin counterstaining for lipid analysis. We scored the liver histology by using a semiquantitative method: steatosis (scored 0–3), lobular inflammation (scored 0–3), and hepatocellular ballooning (scored 0–2), as described in our previous report^9^. We scored three representative areas in each section and used the averages as the final scores. The sum of the scores of steatosis, lobular inflammation, and hepatocellular ballooning was presented as the NAFLD activity score (NAS).

### Analysis of SCFAs in plasma

We performed SCFA analysis for nine analytes. We purchased acetate, propionate, lactate, butyrate, isobutyrate, valerate, isovalerate, pivalate, and hexanoate from Wako Pure Chemical Co. We obtained propionate-*d*_6_, butyrate-*d*_5_, valerate-*d*_9_, and hexanoate-*d*_11_ from Sigma-Aldrich Co. (St. Louis, MO) and CDN Isotopes Co. (Quebec, Canada) for use as internal standards (IS). We purchased triphenylphosphine (TPP), 2,2-dipyridyl disulfide (DPDS), and 2-picolylamine from Tokyo Kasei Co. (Tokyo, Japan). We used methanol to prepare these stock solutions.

We used the Waters Acquity H Class ultra-performance liquid chromatography (UPLC) system (Waters Co., Milford, MA). We performed a reverse phase analysis via an Acquity UPLC BEH C_18_ column (1.7 μm, 2.1 × 100 mm) at 40 °C, with an injection volume of 5 μL. The mobile phase, which contained solvent A (0.1% formic acid in water) and solvent B (0.1% formic acid in methanol), was delivered at a 0.3 mL/min flow rate. We used the following gradient elution: B% = 2, 2, 35, 45, and 98 (0, 3, 10, 12, and 14 min). We operated the Waters Xevo TQD triple quadrupole mass spectrometer with an electrospray ionization (ESI) source in the positive mode, with the following ionization source conditions: capillary voltage, 2.00 kV; cone voltage, 20–70 V; collision energy, 10–40 eV; source temperature, 150 °C; and desolvation temperature, 400 °C. The cone gas flow was 50 L/h and the desolvation gas flow was 800 L/h, and they were obtained via a nitrogen source (N_2_ Supplier Model 24 S; Anest Iwata, Yokohama, Japan). In view of a previous report describing derivatization of carboxylic acids^59^, we used methanol to dilute mixed SCFAs and IS solutions. We reacted these solutions with 2-picolylamine in DPDS and TPP in acetonitrile for 10 min at 60 °C. We removed the reaction mixtures and re-dissolved them in 100 μL of methanol/water (80:20, v/v). We used UPLC-ESI-MS/MS to analyze the derivatization solutions (5 μL). We added thawed plasma samples to IS and mixed these samples with equal volumes of methanol and QuEChERS (Supel QuE PSA (EN) 25 mg), after which they were vortexed vigorously and centrifuged for 5 min at 15,000 rpm. We then removed the supernatant and re-dissolved the remaining residue in methanol and derivatized it by using the process described above for 2-picolylamine. We then used UPLC-ESI/MS/MS to analyze the samples.

### Statistical analysis

The statistical differences in the body weight, visceral fat, the ratio of Firmicutes to Bacteroidetes, and plasma SCFAs between groups were evaluated using one-way analysis of variance followed by Dunnett’s test (Fig. 2a,c, Fig. 4b, and Fig. 5), by means of Prism software (GraphPad Software, La Jolla, CA). We set the level of significance at *P* < 0.05. In order to assess whether significant differences occurred in an abundance of bacteria, a multiple-comparison procedure that controls the false-discovery rate (FDR) were performed by using the Benjamini and Hochberg method. FDR adjusted *Q* value of 0.05 was considered statistically significant (Fig. 3, Fig. 4a, and Table 1).
